# Supplementary material for: The extinction time under mutational meltdown driven by high mutation rates
Source: Ecol Evol. 2022 Jul 6;12(7):e9046. doi: 10.1002/ece3.9046 (PMC9257376; doi:10.1002/ece3.9046)
Supplement: Supplementary file 1 — Appendix S1‐S4 [file ECE3-12-e9046-s001.pdf]

Appendix S1 A smooth approximation of Gessler's ratchet speed

Gessler (1995) approximated the rate of Muller's ratchet (in particular, the rate of the loss of the fittest class) as

$$v_R = s(2k - b), \tag{13}$$

where

$$k := \min_{x\in\mathbb{N}} \left\{ x \text{ such that } \frac{Ne^{-\theta}\theta^x}{x!} - 1 \geq 0 \text{ and } x \leq \theta \right\}, \tag{14}$$

$$b := \min_{x\in\mathbb{N}} \left\{ x \text{ such that } \frac{N\lambda^{-(x-k)}}{(x-k)!} \left(e^{\lambda} - \sum_{i=0}^{x-k-1} \frac{\lambda^i}{i!} \right)^{-1} - 1 \geq 0 \text{ and } k \leq x \leq \theta \right\}, \tag{15}$$

for $\theta := \mu(1-s)/s$ and $\lambda := \theta - k$. Here N denotes the total population size, which Gessler assumed to be constant. In our model this translates to $N = K$, as our population remains at its carrying capacity during the ratchet phase.

For simplicity let us define

$$f_k(x) := \frac{Ne^{-\theta}\theta^x}{x!} - 1, \quad x \in \mathbb{N} \tag{16}$$

and

$$f_b(x) := \frac{N\lambda^{-(x-k)}}{(x-k)!} \left(e^{\lambda} - \sum_{i=0}^{x-k-1} \frac{\lambda^i}{i!} \right)^{-1} - 1, \quad x \in \mathbb{N}, \tag{17}$$

which appear in the definitions of the value k in (14) and b in (15).

Since $k, b \in \mathbb{N}$, Gessler's speed of the ratchet, eq. (13), can be non-monotonous with respect to the parameters, as shown in Figure 2. For instance, both coefficients k and b are monotonous and non-decreasing functions of the mutation rate μ. However, the following situation might occur:

$$k(\mu + \delta) = k(\mu) \text{ and } b(\mu + \delta) > b(\mu), \text{ for some } \delta > 0, \tag{18}$$

i.e., with increasing μ, b may take on a larger value while k remains constant, which results in a sudden decrease of the ratchet speed (which should monotonously increase with the mutation rate),

$$v_R(\mu + \delta) < v_R(\mu). \tag{19}$$

To avoid this behavior, we extend the domain of the functions f_k, f_b as defined in (16)-(17), to

positive real numbers. We define

$$\overline{f_k}(x) := \frac{Ne^{-\theta}\theta^x}{\Gamma(x+1)} - 1, \quad x \in [0, \infty), \tag{20}$$

where we use the Euler Gamma function Γ which generalises the factorial operation over the real numbers (Abramowitz and Stegun 1972). In particular, $\overline{f_k}(x) = f_k(x)$ for all $x \in \mathbb{N}$.

The extension of the function $f_b(x)$ in (17) to positive real numbers needs more care because x appears in the upper bound of summation of $\sum_{i=0}^{x-k-1} \frac{\lambda^i}{i!}$. Defining $n_{x,k} := \lfloor x - k - 1 \rfloor \in \mathbb{N}$, i.e. $n_{x,k} \leq x - k - 1 < n_{x,k} + 1$, the function

$$\overline{g}(x-k-1) := \sum_{i=0}^{n_{x,k}} \frac{\lambda^i}{i!} + \big((x-k-1) - n_{x,k}\big)\frac{\lambda^{n_{x,k}+1}}{(n_{x,k}+1)!}, \quad x-k-1 \in [0, \infty) \tag{21}$$

represents an interpolation of $g(x-k-1) := \sum_{i=0}^{x-k-1} \frac{\lambda^i}{i!}$ on $[0, \infty)$. Indeed, when $x-k-1 \in \mathbb{N}$, equation (21) corresponds to the original sum, and $\overline{g}(n) = g(n)$ for all $n \in \mathbb{N}$. Therefore, the following function extends f_b to positive real numbers:

$$\overline{f_b}(x) := \frac{N\lambda^{-(x-k)}}{\Gamma(x-k+1)} \left(e^{\lambda} - \sum_{i=0}^{n_{x,k}} \frac{\lambda^i}{i!} - \big((x-k-1) - n_{x,k}\big)\frac{\lambda^{n_{x,k}+1}}{(n_{x,k}+1)!} \right)^{-1} - 1, \quad x \in [k, \theta). \tag{22}$$

Since both f_k and f_b are increasing functions of $x \in \mathbb{N}$, the problem of finding the minimum integers k, b such that $f_k(k), f_b(b) \geq 0$, as required in equations (14) and (15), easily extends to the problem of finding the zeros of $\overline{f_k}$ and $\overline{f_b}$.

Therefore, if there exist $\hat{k} \in [0, \theta]$ and $\hat{b} \in [k, \theta]$, such that $\overline{f_k}(\hat{k}) = \overline{f_b}(\hat{b}) = 0$, the following expression provides a continuous extension of the original Gessler's ratchet speed:

$$\overline{v_R} = s(2\hat{k} - \hat{b}). \tag{23}$$

Similar to the original formula by Gessler (1995), the above expression is well defined only for suitable choices of the parameters μ, s, N.

Figure 2 in the main text compares the ratchet speed in its original discrete version by Gessler (1995) with our smooth monotonic extension.

Appendix S2 Description of the computational analysis

The simulation of the population dynamics and mutation accumulation was performed using the script 'simulate_mutational_meltdown.jl'. First, the parameters are initialised and a founder

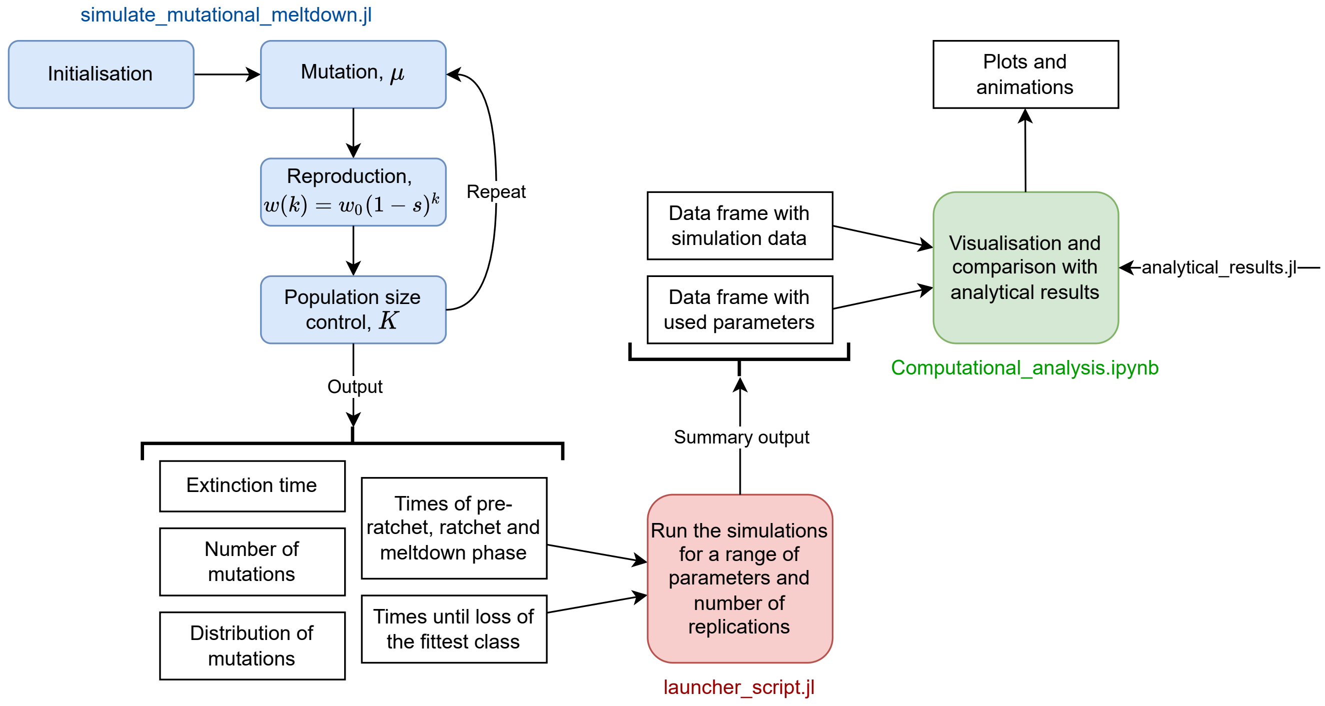

Figure S1: Work flow of the computational analysis

population is created. At each new time point, the population is updated in three steps: (1) in the *Mutation* step each individual acquires new mutations $\sim$ Poi(μ)); (2) in the *Reproductive* step each individual is replaced by offspring $\sim$ Poi $\left(w_0(1-s)^k\right)$); (3) in the *Population-size control* step, if the population has grown over its carrying capacity, randomly selected individuals are removed until population size is reduced to K individuals. Possible outputs are the extinction time, the number or the distribution of mutations every generation, the beginning and end time points of the pre-ratchet, ratchet and meltdown phases, and the times until the fittest classes are lost.

We ran the simulations for the parameter regime described in the manuscript using the script 'launcher_script.jl'. Here, we were interested in the times of pre-ratchet, ratchet and meltdown phase and the times until loss of the fittest class. The output is a data frame that contains the simulation output and data frame that contains the parameters used for this specific simulation. Each specific simulation can be identified and reproduced via a seed from the random number generator.

The analysis was performed in the Jupyter notebook 'Computational_analysis.ipynb', and the simulations were implemented in the programming language 'julia', version 1.6 (Bezanson et al. 2017; Besançon et al. 2021).

Appendix S3 Power law relationship between extinction time and mutation rate

We noticed in Section 3.4 that the extinction time decays as a power law of the mutation rate, i.e. $T_E \approx \alpha\mu^{-\beta}$, see Figure S2 for the numerical estimates of the scaling factor α and the power-law exponent β using ordinary least squares regression.

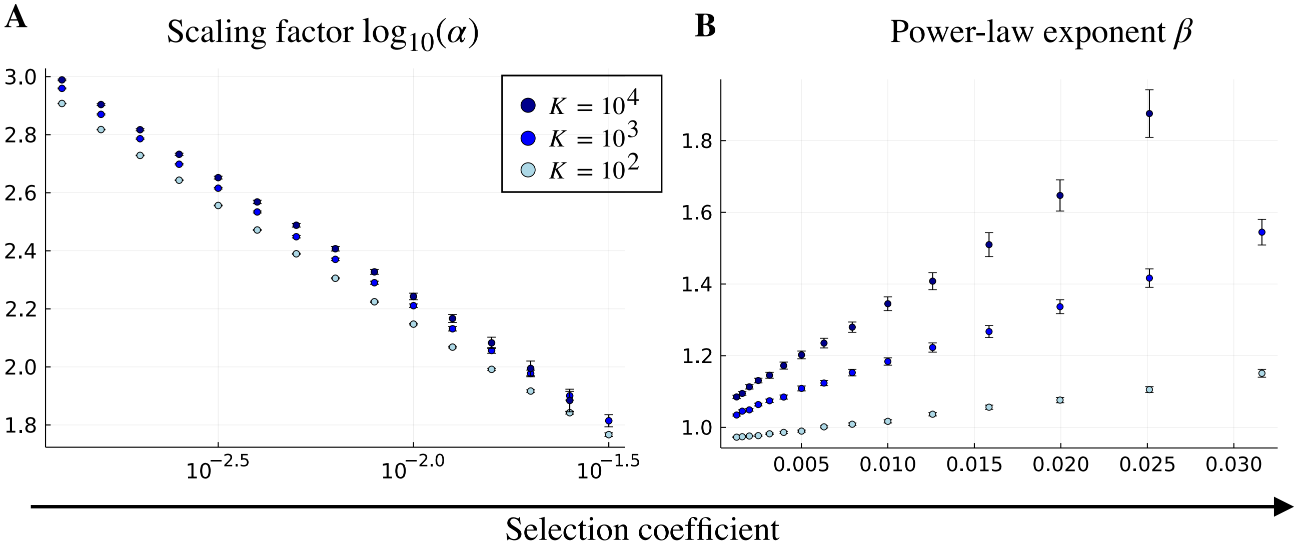

Figure S2: *Numerical estimation of the power law relationship between the mean extinction time and the mutation rate* $T_E \approx \alpha\mu^{-\beta}$. We used ordinary least squares regression to estimate (A) the scaling factor α and (B) the power-law exponent β and the respective standard errors depending on the selection coefficient s and for different carrying capacities K.

Appendix S4 Simulation data for carrying capacities K=100, 10000

In addition to the simulation data presented in Section 3.6 with a carrying capacity of $K = 1000$, we also performed simulations for carrying capacities of $K = 100$ and $K = 10000$, see Figures S3 and S4. They showed similar results and are consistent with our expectation on the dependence of the extinction time on the carrying capacity as described in Section 3.4, also shown in Figure 3B.

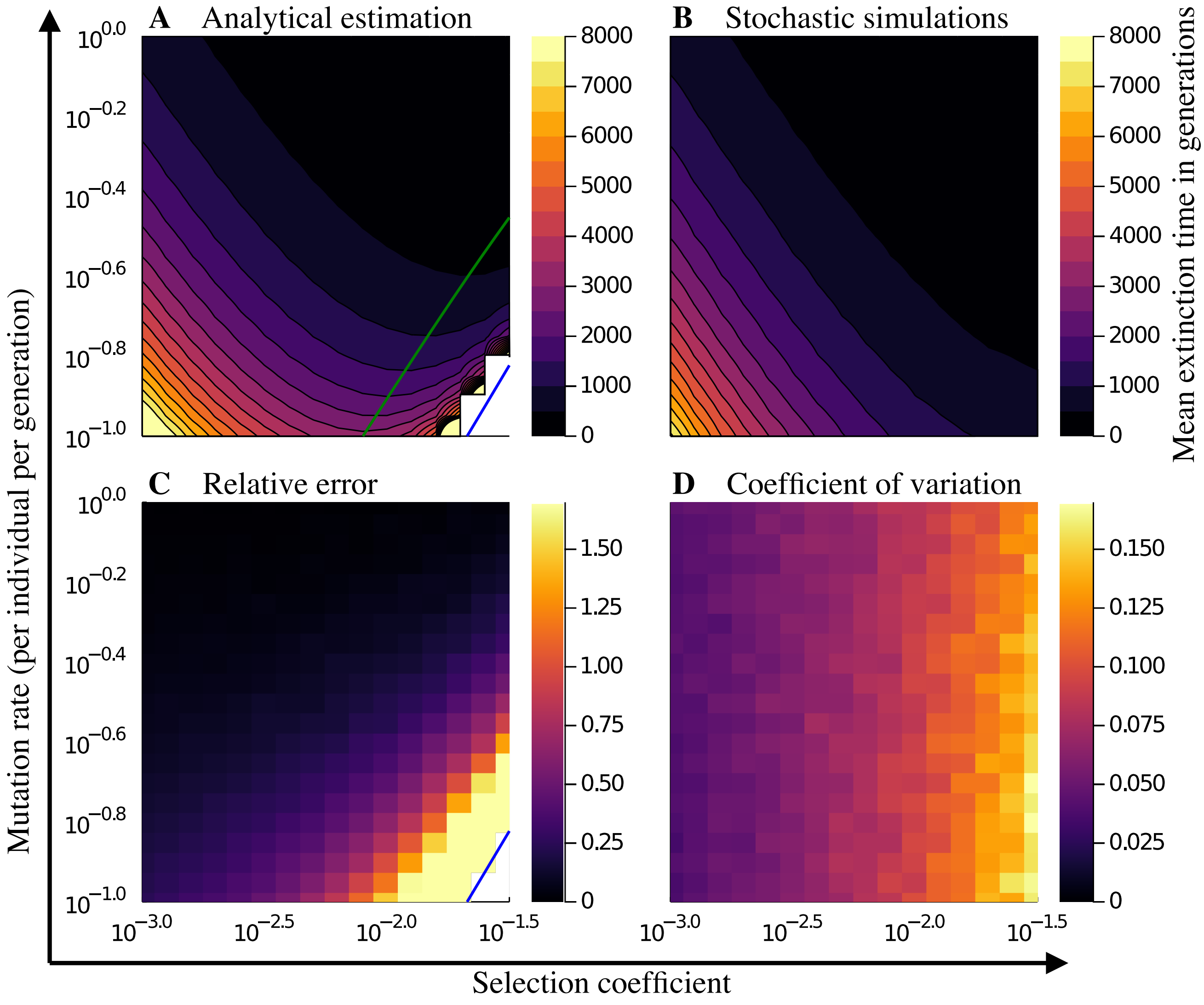

Figure S3: Carrying capacity $K = 100$: *Comparing the analytically predicted mean extinction time with results from stochastic simulations for a range of mutation rates and selection coefficients.* **A, B** The contour lines give parameter combinations with equal mean extinction time. Our analytical estimation is not applicable and returns an infinite extinction time if the mutation-selection balance is stable, which is indicated by the blue line in panel A. This is not observed in stochastic simulations shown in panel B. The predicted optimal selection coefficient for which the extinction time is minimal (green line panel A) is smaller than the one observed in stochastic simulations. **C** The relative error of our analytical estimation is small in general but becomes large as the boundary of our parameter regime is approached. **D** The coefficient of variation of the extinction time in the simulations is comparably small, but positively correlated with the selection coefficient. (Other parameters: founder population size $N_0 = 20$, wild-type reproduction rate $w_0 = 2$ and $n = 150$ simulation runs.)

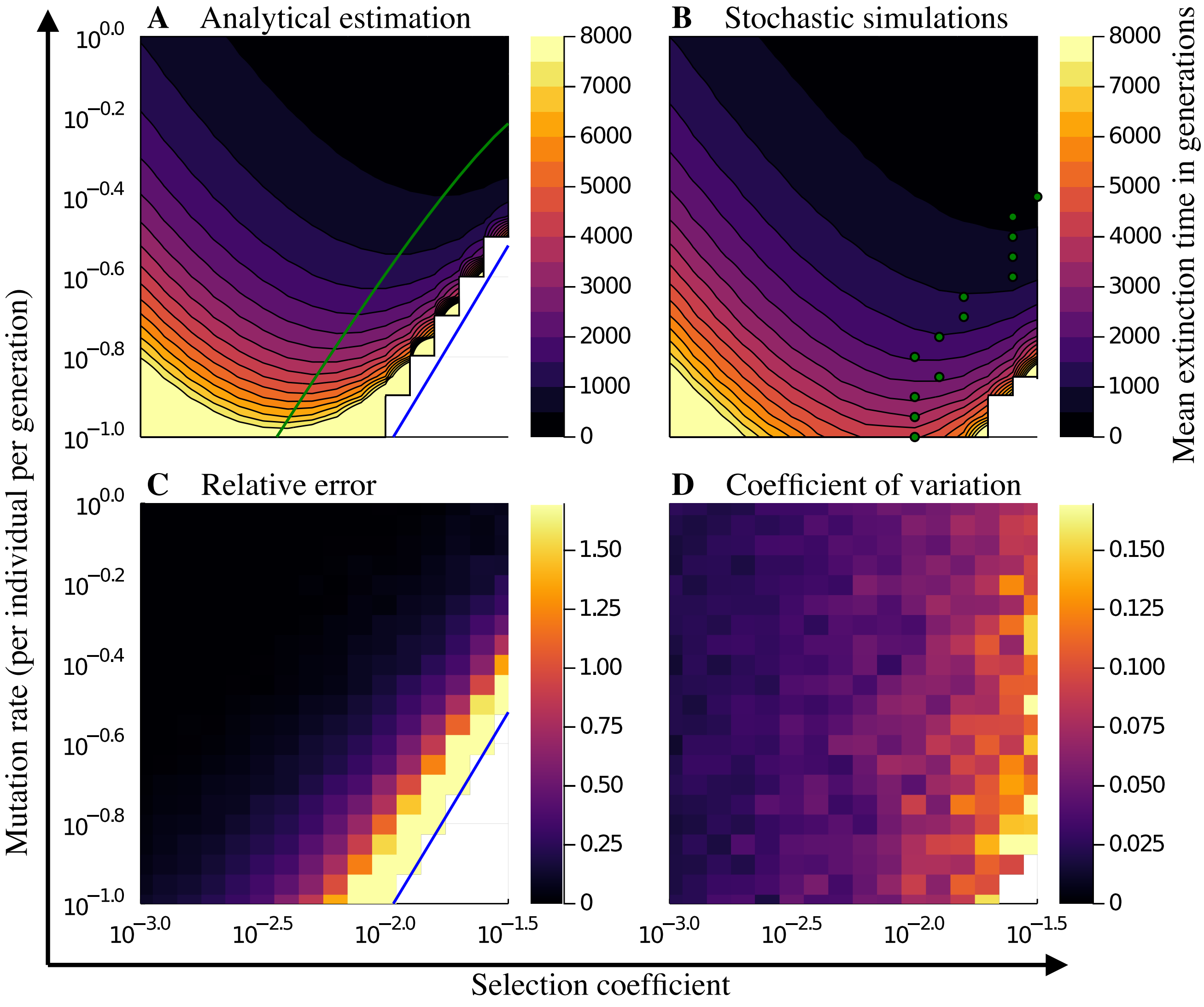

Figure S4: Carrying capacity $K = 10000$: *Comparing the analytically predicted mean extinction time with results from stochastic simulations for a range of mutation rates and selection coefficients.* **A, B** The contour lines give parameter combinations with equal mean extinction time. Our analytical estimation is not applicable and returns an infinite extinction time if the mutation-selection balance is stable, which is indicated by the blue line in panel A. This is not observed in stochastic simulations shown in panel B. The predicted optimal selection coefficient for which the extinction time is minimal (green line panel A) is smaller than the one observed in stochastic simulations (green dots panel B). **C** The relative error of our analytical estimation is small in general but becomes large as the boundary of our parameter regime is approached. **D** The coefficient of variation of the extinction time in the simulations is comparably small, but positively correlated with the selection coefficient. (Other parameters: founder population size $N_0 = 20$, wild-type reproduction rate $w_0 = 2$ and $n = 20$ simulation runs.)

References

Abramowitz, M., & Stegun, I. A. (1972). Handbook of mathematical functions with formulas, graphs, and mathematical tables (Vol. 55) *US Government printing office*.

Besançon, M., Papamerkou, T., Anthoff, D., Arslan, A., Byrne, S., Lin, D., & Pearson, J. (2021). Distributions.jl: Definition and modeling of probability distribution in the JuliaStats ecosystem. *Journal of Statistical Software, 98,* 1-30.

Bezanson, J., Edelman, A., Karpinsky, S., & Shah, V. B. (2017). Julia: A fresh approach to numerical computing. *SIAM Review, 59,* 65-98.

Gessler, D. D. G. (1995). The constraints of finite size in asexual populations and the rate of the ratchet. *Genetical Research, 66,* 241-253.
